# Leaching of Major and Minor Elements during the Transport and Storage of Coal Ash Obtained in Power Plant

**DOI:** 10.1155/2014/212506

**Published:** 2014-06-29

**Authors:** Rada Krgović, Jelena Trifković, Dušanka Milojković-Opsenica, Dragan Manojlović, Jelena Mutić

**Affiliations:** ^1^RTB Kolubara, Svetog Save 1, Lazarevac, Serbia; ^2^Faculty of Chemistry, University of Belgrade, Studentski Trg 12-16, P.O. Box 51, 11158 Belgrade, Serbia

## Abstract

In power plant, coal ash obtained by combustion is mixed with river water and transported to the dump. Sequential extraction was used in order to assess pollution caused by leaching of elements during ash transport through the pipeline and in the storage (cassettes). A total of 80 samples of filter ash as well as the ash from active (currently filled) and passive (previously filled) cassettes were studied. Samples were extracted with distilled water, ammonium acetate, ammonium oxalate/oxalic acid, acidic solution of hydrogen-peroxide, and a hydrochloric acid. Concentrations of the several elements (Al, As, Cd, Co, Cu, Cr, Fe, Ba, Ca, Mg, Ni, Pb, and Zn) in all extracts were determined by inductively coupled plasma atomic emission spectrometry. Pattern recognition method was carried out in order to provide better understanding of the nature of distribution of elements according to their origins. Results indicate possible leaching of As, Ca, Cd, Cu, Zn, and Pb. Among these elements As, Cd, and Pb are toxicologically the most important but they were not present in the first two phases with the exception of As. The leaching could be destructive and cause negative effects on plants, water pollution, and damage to some life forms.

## 1. Introduction

Coal ash is the fossil-fuel combustion residue from coal power plants. Deposits of fuel ashes as a source of pollution in environment could be a serious problem [1]. Coal combustion in power plants generates large amounts of ash that is marginally used or, in most cases, stored more or less unprotected in the environment where it can represent a significant source of heavy metals, PAHs, and other pollutants [2].

A series of physicochemical transformations take place during the coal combustion in the power plants and often can change solubility and association patterns of various elemental species. After coal combustion, coal ash is usually stored in huge coal ash dumps, either in dry or wet state. Storage of wet coal ash usually protects environment from wind spreading but decreases the time necessary for leaching of various elements [3].

The environmental impact of coal ash production has at least two aspects: emission and deposition of enormous amounts of coal ash, polluting air, water, and soil and leaching of microelements (including toxic heavy metals) but also major cations and anions from ash by atmospheric and surface waters. During the coal combustion in power plants, only organic part of coal burns, while the inorganic components mainly remain in the ash as by-product. Inorganic components of coal as well as many trace elements were concentrated in the ash [4].

In the literature various extraction procedures for soil and sediments were reported. They were based on different sequence schemes and various operating conditions [5–8]. The use of sequential techniques provides researchers with essential information on the composition of materials as well as the mobility of metals through environment [9]. Also, various extraction procedures for the analysis of fly ash were described [10–16].

Thermoelectric power plant (TPP) “Kolubara” is the oldest active plant in the system of the electric power distribution of Serbia commissioned in 1956. With its five units, with total installed power of 270 megawatts, at that time it was the largest power facility in the country. Together with some other power plants it produces electricity for Belgrade area and most of Serbia. “Kolubara” power plant produces 1.1 × 10^9^ kWh annually as well as 6–8 × 10^9^ kg of coal ash. In that sense, the aim of this paper is to determine the impact of leaching of minor and major elements on the environment during and after the transport of coal ash to dumps (active cassette) as well as direct deposition to soil (passive cassette) in order to obtain detailed data on coal ash and leaching of various elements from it.

## 2. Material and Method

### 2.1. Reagents

All chemicals were analytical grade and supplied by Merck (Darmstadt, Germany) or Sigma Aldrich (Taufkirchen, Germany). High-purity water (resistivity >16 MΩcm) produced by reverse osmosis and demineralization was used. Standard solutions were prepared from multielement standard stock solutions (Merck, 1000 mg/L). These multielement standards and blanks were prepared in the same matrix as the extracting reagents in order to minimize the matrix effects. The laboratory glassware and plastic ware were cleaned by immersing the vessels in 6 mol/L HNO_3_ overnight and then washed successively with deionized water.

### 2.2. Samples

Samples of filter ash were collected from electrofilter (EF) in power plant “Kolubara” (Veliki Crljeni, Serbia). 0.5 kg sample was prepared from 5 kg of air-dried sample by “quartering” method. Twenty 1 g portions of 0.5 kg sample were analysed [17].

Filter ash from power plant was mixed with river water and transported to active cassette. Ten kilograms of “wet” filter ash from active cassette (A) was sampled and air-dried at room temperature for 3 weeks. Then by the quartering method 0.5 kg sample was obtained and 20 portions were analysed.

The active cassette becomes passive cassette after complete filling which can last up to ten or more years. Passive cassette was near Lazarevac, Serbia. It has an area of 4 km^2^ and coordinates 44°  29′  25′′ N and 20°  19^′  ^6′′ E. Sampling site at passive cassette has an area of 1 ha (100 × 100 m). The samples from passive cassette were taken at two depths: 30 cm to 50 cm (PA) and 50 cm to 80 cm (PB). Sampling was performed by the principle of diagonal grid sampling and 1 kg of soil was taken from ten points at two depths covering the entire surface of the cassette [17]. Composite samples of ten soil subsamples were air-dried at room temperature for three weeks. The same quartering method was used to obtain 0.5 kg sample and 20 portions were taken (20 for PA and 20 for PB).

### 2.3. Sequential Extraction Procedure

A five-step sequential extraction procedure for coal ash was applied [4]. The following solutions were used for sequential extraction of a portion (1 g air-dried) of each sample.

- Distilled water (the weakest extractant). The sample was transferred to an Erlenmeyer flask and 30 mL distilled water was added. The mixture was shaken for 6 h at ambient temperature in a horizontal rotary mechanical shaker at a speed of 200 rpm. The extract was separated from the solid residue by centrifugation at 3000 rpm for 5 min and decantation of the supernatanat liquid into a glass. The solid residue was retained for the next step.
- Ammonium-acetate, concentration of 1 mol/L. The residue was shaken with 30 mL of ammonium-acetate solution for 6 h and then centrifuged.
- Ammonium oxalate, concentration of 0.2 mol/L, and oxalic acid, concentration of 0.2 mol/L. The extraction was performed by adding 30 mL of solution, shaking for 10 h, and then centrifuging. The use of ammonium oxalate for the extraction procedure causes the analytical problems due to the crystallization. In order to overcome these interferences samples are diluted with distilled water 1 : 5 (v/v) or another type of nebulizer was used, Burgener miramist nebulizer (Thermo Scientific), with enhanced parallel path design that can handle high level of particulates in samples.
- Hydrogen-peroxide, 30% solution, adjusted to pH 2 with concentrated HNO_3_. 10 mL was added and heated on a water bath at 85°C in order to dissolve organic/sulfide matter. Then 30 mL of 3.2 mol/L ammonium-acetate solution was added and shaken 30 min in order to desorb eventually readsorbed elements.
- The residue was treated 5 h with 10 mL of 6 mol/L HCl and solution in the flask was covered with watch glass at the water bath (85°C). Then the liquid in the flask was evaporated. To the dry cooled residue 5 mL of 6 mol/L HCl solution was added, heated for 4 h at the temperature of 85°C, and centrifuged.

After each extraction step all residues were washed with distilled water; the combined extracts and washings were analyzed. Also, all solutions collected during sequential extraction were filtered through medium quantitative filter paper before the analysis.

The sequential extraction procedure validated using certified reference material ERM-CC141 (loam soil) JRC-IRMM, Belgium, obtaining recoveries with deviation below 5% from certified values. The results for each element obtained by the treatment with aqua regia were equal (within the error limits) to the corresponding reference values “(see Table S8 in Supplementary Material available online at http://dx.doi.org/10.1155/2014/212506)”.

For the extraction, a horizontal, mechanical water bath shaker was employed. A centrifuge was used for separation of the liquid phase from the precipitate.

### 2.4. Instrumentation

The concentrations of the metals from all phases of sequential extraction were determined by inductively coupled plasma atomic emission spectrometry (Thermo Scientific, United Kingdom), model 6500 Duo, equipped with a CID86 chip detector. This instrument operates sequentially with both radial and axial view of plasma. The entire system was controlled with Iteva software. The wavelengths were 396.1 nm (Al), 189.0 nm (As), 455.4 nm (Ba), 393.3 nm (Ca), 214.4 nm (Cd), 228.6 nm (Co), 283.5 nm (Cr), 324.7 nm (Cu), 259.9 nm (Fe), 279.5 nm (Mg), 221.6 nm (Ni), 220.3 nm (Pb), and 213.8 nm (Zn).

### 2.5. Data Analysis

Principal component analysis (PCA) has been performed by means of PLS ToolBox, v.6.2.1, for MATLAB 7.12.0 (R2011a). PCA was carried out as an exploratory data analysis by using a singular value decomposition algorithm (SVD) and a 0.95 confidence level for *Q* and *T* ^2^ Hotelling limits for outliers. Using only a limited number of principal components (PCs), the dimensionality of the retention data space was reduced, further analysis was simplified, and the parameters were grouped according to similarities.

Descriptive statistics and Kruskal-Wallis one-way analysis of variance by ranks test have been performed by means of a demo version of NCSS statistical software [18].

All data were pretreated (mean-centered and scaled to the unit standard deviation) before any statistical operations in order to prevent highly abundant components to dominate in the final result over the components present in much smaller quantities.

## 3. Results and Discussion

The summarized parameters of descriptive statistics obtained from the metal content analysis of filter ash, and ash from active (currently filled) and passive (previously filled) cassettes, after five extraction steps, are presented in Tables S1–S4. The contents of thirteen elements of coal ash by phases are presented in Figure S1.

Transporting water was taken from “Kolubara” river and controlled quarterly. A part of the results relating to the elements in the water were presented in Table S5.

**Figure 1:**
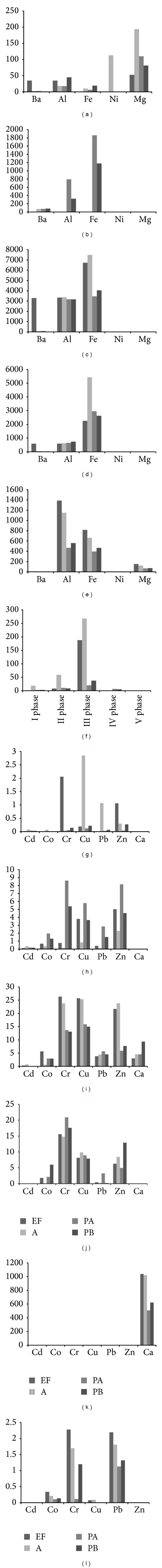
The content of twelve elements of coal ash: (a–e) macroelements (Ba, Al, Fe, Ni, and Mg) for each phase of extraction, respectively; (f) content of As by phases; (g–l) micro and trace elements (Cd, Co, Cr, Cu, Pb, Zn, and Ca) for each phase of extraction, respectively; and (k) content of Ca in fifth phase.

In addition, in order to facilitate the perception of the multivariate data, the mean values of content of each metal are presented in Figure 1. The content of macroelements (Ba, Al, Fe, Ni, and Mg) is shown on graphs (a–e), and the content of micro elements (Cd, Co, Cr, Cu, Pb, Zn, and Ca) is shown on graphs (g–l), for each phase separately.  Figure 1(f) represents the content of As by phases. Filter ash samples were collected during one day but for some elements high variability among the data was found (Table S1). This is particularly pronounced in the first phase of extraction for elements such as As, Al, Ba, Cu, and Zn. For example, content of Al in first phase of the first depth was 17.56 mg/kg and SD was 10.24 mg/kg. This might be an indication that the concentration of Al varies significantly within this depth. A high variability among the data of the content of Al in passive cassette, second depth, was also observed (44.89 mg/kg, SD 41.02 mg/kg). The differences could be attributed to various compositions of coal combusted in power plant and to changes in atmospheric conditions during long time intervals. It is convenient to expect the heterogeneity of a huge number of real samples. Samples from passive cassette were taken from depth 30 to 50 cm and 50 to 80 cm in order to avoid possible changes which could be expected if they had been collected at the surface.

The highest amounts of elements were extracted in third (reducible) and fourth (organic-sulphide) phases of extraction, respectively (Figures 1(c) and 1(i)).

The first phase of the extraction is characterized by high amount of Mg, especially in the active cassette (Figure 1(a)). Namely, coal ash obtained by combustion might be mixed with water and transported to the dump. Samples from passive cassette have a higher content of Mg compared to samples from filter ash. It could be explained by periodical washing of pipelines by hydrochloric acid which dissolves precipitated carbonates. Magnesium is also extracted in the fifth phase with the slightly higher content in samples from filter ash and active cassettes. The first phase was also characterized with high amount of Ni extracted from samples in active cassettes (Figure 1(a)). In all other extraction phases Ni was present in very small amount only in samples from passive cassette. Calcium was extracted only in the fifth phase, with higher amount in samples from filter ash and active cassettes (Figure 1(k)) which indicates that Ca was bound to acid soluble residue such as detritus silicates and crystalline Fe-oxide [19]. Lower content of Ca in passive cassette indicates its leaching during transport. However, its emission has no toxicological importance. The third phase of extraction was characterized with very high amount of Fe, particularly in samples from active cassettes and filter ash (Figure 1(c)). Iron was also present in significant amount in the fourth phase, in samples from active cassettes, and in second phase, only in samples from passive cassette (Figures 1(d) and 1(b), resp.). This could be attributed to the reducing conditions in passive cassette where the iron was reduced and therefore became more available than in active cassettes. The highest content of Al was detected after the third extraction, in almost equal amounts in samples from all cassettes (Figure 1(c)). Significant amount of this element was also found after the fifth extraction, especially in samples from filter ash and active cassettes (Figure 1(e)). It was also found after second extraction, in samples from passive cassette, especially from first depth (Figure 1(b)). Samples from filter ash after the third, fourth, and first phases of extraction were characterized with high amount of Ba (Figures 1(a), 1(c), and 1(d)). Lower content of Ba in all other cassettes suggested it leaches during the transport but without any environmental pollution. Significant amount of As in samples from active cassettes was also found during the first, second, and third extraction phases (Figure 1(e)). Arsenic is volatile; during the combustion of coal it could be found at ash particles as the result of condensation. Such arsenic is on the surface and it could be easily extracted. A considerable portion of arsenic originates from transporting water to active cassettes and contributes to high amount of arsenic in the first phase (see Table S5). The content of As in samples of filter ash and active cassette is considerably higher than in passive cassette. This could be attributed to their continual leaching to the environment that could be toxicologically important. Considering its high toxicity as well as high production of ash it could be a serious environmental problem. The contents of minor and trace elements (Cr, Cu, Pb, Zn, and Co) are usually higher in samples from filter ash and active cassettes compared to samples from passive cassette, indicating their leaching (Figures 1(g)–1(j), and 1(l)) which could be a serious problem concerning their toxicity. Cadmium was present in samples in the smallest amount compared to all other elements. The highest content was observed for second extraction phase. Wadge and Hutton [20] found that 20% of total Cd in coal ash was in the exchangeable fraction (phases I and II). The extraction data indicate the mobility of Cd which depends on sample crystallinity. Austin and Newland [21] showed that Cd is a typical surface-adsorbent element in the combustion process.

For the statistical evaluation of the observed differences in the metal content, the Kruskal-Wallis test was employed for each of the metals separately, as variables, taking the type of cassette as a single factor, for each phase of the extraction. The results are presented in Table S6. In the cases of the observed a statistically significant difference between the medians, Kruskal-Wallis multiple-comparison *Z*-value test was performed. Cassettes with different content of a given metal are denoted in parentheses (Table S6). A statistically significant difference between metal content of samples from different origins could be observed in almost every phase of extraction.

**Figure 2:**
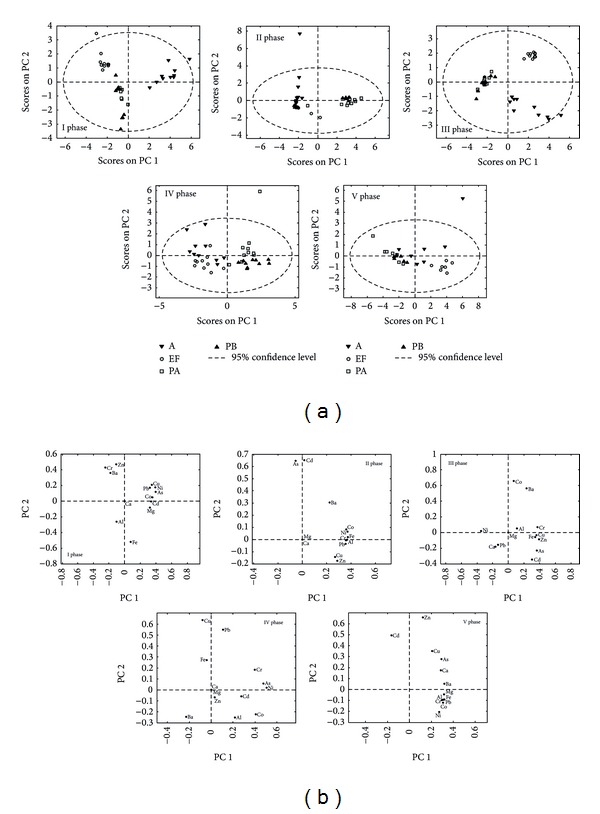
PCA, (a) score plots for each phase of extraction, respectively; (b) loading plots for each phase of extraction, respectively.

In order to get the basic insight into the data structure and identify similarities and specific grouping patterns, PCA has been carried out on metal content data, for each phase separately. Statistical parameters (number of principal components (PCs) and percent variance captured by first three PCs) for five PCA models are presented in Table  S7. Mutual projections of factor scores and their loadings for the first two PCs, of five PCA models, have been presented in Figure 2.

PCA for the first phase of extraction resulted in a model which explains 77.41% of total variance. Score plot (Figure 2(a)) reveals three distinct groups of samples belonging to filter ash, active cassette, and two types of passive cassette. The loading plot (Figure 2(b)) implies that the most influential metals discriminating between samples from filter ash and those from active and passive cassette are Zn, Cr, and Ba. The major factors that lead to the separation of samples from active cassette from the other types are Cu, Pb, Ni, As, Co, Cd, and Mg, since they show a high positive impact alongside the PC1 direction. The content of Al and Fe separates samples from passive cassette. Therefore, it would be reasonable to expect higher values of Zn, Cr, and Ba in filter ash samples, higher values of Cu, Pb, Ni, As, Co, Cd, and Mg in active cassette samples, and higher amount of Al, that is, Fe in passive cassette samples. The content of Ca does not significantly contribute to either PC1 or PC2, and it should not be taken as a significant criterion when comparing samples of different origins.

PCA for the second phase of extraction resulted in a model which explains 92.33% of total variance. Score plot (Figure 2(a)) reveals two groups of samples, distinguished alongside the PC2 direction. One group consisted of samples from filter ash and active cassette and four samples from PA. Also, within this group, two subgroups existed: one is situated in the upper left part of the graph and comprises samples from active cassette and the other in the lower left part, with samples from filter ash. The other main group consisted of samples from two types of passive cassette. Loading plot implies that As and Cd are parameters that have the most positive impact on PC2 direction. The content of Co, Ni, Fe, Cr, Al, and Pb significantly affects the PC1 component in a positive manner. Higher values of As and Cd content in active cassettes could be expected (Figures 2(a) and 2(b)). Their high concentrations in this phase indicate that they can be potential pollutants. Also, the content of Ba should be higher in samples from active and passive cassettes compared to filter ash. The content of Ca and Mg does not significantly contribute to the model.

PCA for the third phase of extraction resulted in a model which explains 94.42% of total variance. Score plot (Figure 2(a)) reveals three distinct groups of samples belonging to filter ash, active cassettes, and two types of passive cassette. The major factors that lead to the separation of samples from active cassette from the other types are As and Cd. The content of Co and Ba separates samples from filter ash, while Ni content distinguished samples from passive cassette. The content of Mg should not be taken as a significant criterion when comparing samples of different origins.

Differences between four origins are less pronounced in fourth and fifth phases of extraction (Figure 2(a)). Still, two principal components differentiate samples from passive cassette from those in filter ash and active cassette. PCA for the fourth and fifth phases of extraction resulted in a model which explains 60.49% and 94.02% of total variance, respectively. In fourth phase, the major factors that lead to the separation of samples from active cassette are Fe, of filter ash samples are Ba, and of samples from passive cassette (first and second depth) are Pb and Co, respectively (Figure 2(b)). In fifth extraction phase all metals were extracted in higher amount in samples from filter ash and active cassette compared to samples from passive cassette (Figure 2(b)).

Similarities in the content of all elements in all phases for passive cassette are evident and they corroborate conclusion based on Figure 1 and Tables S1–S4.

The first two phases (adsorbed and ion-exchangeable) of sequential extraction are environmentally accessible. Some of the changes in samples could be observed. Zn, Cr, and Ba were present in EF in adsorbed form, while changes in ionic strength of transportation water affect Cu, Pb, Ni, As, Co, Cd, and Mg leaching which were in ion-exchangeable forms. The noticeable fraction of Zn, Cr, and Cu in filter ash and active cassette associated with oxide of iron and manganese was dissolved in the third phase (Figure 1(i)). Contrary, these metals were found in the first two easily exchangeable phases in passive cassette (Figures 1(h) and 1(g)). It means that under reduction conditions and lower pH, Cr, Cu, and Zn migrated from easily reducible fraction to adsorbed and ion-exchangeable form [22, 23]. Chromium also was associated with organic-sulphide fraction as well as with aluminosilicate in V phase in all samples.

In all samples Al and Fe with the same content were associated with oxide of iron and manganese in reducible phase and organic-sulphide fraction. Only in samples from passive cassette Al and Fe extracted in ion-exchangeable phase indicated that they were sensitive to the change of reduction conditions, temperature, and presence of anaerobic bacteria. Leaching of lead from samples from filter ash, active cassette and passive cassette was indicated by its distribution. The concentration of adsorbed magnesium is bigger in active cassette than in filter ash proving that river water influences its leaching. As it has been expected, a small amount of magnesium was concentrated in the acid soluble residue (Figures 1(a) and 1(e)). It is known that metals associated with the acid soluble residue are less bioavailable than the metals associated with nonresidual fractions.

## 4. Conclusions

Filter ash samples were collected during one day but for some elements high variability among the data was found. This is particularly pronounced in the first phase of extraction for elements such as As, Al, Ba, Cu, and Zn. Presence of As, Cd, Ni, Pb, Fe, and Mg at least could originate from river water used for transport of ash. Total content of Ba and Co was higher in EF than in active cassette, which could be due to loss during the transportation. Some of the most important migration and changes in samples could be observed. Zn, Cr, and Ba were present in EF in adsorbed form, while changes in ionic strength of transportation water affect Cu, Pb, Ni, As, Co, Cd, and Mg leaching.

The content of elements in passive cassette was not dramatically changed with depth although the filling of these cassettes lasts several years. Small differences between two depths in passive cassette could be attributed to different composition of coal combusted in power plant and to changes of atmospheric conditions. On the other hand, the samples were not taken from the surface where drastic changes in the composition of the passive cassette may occur under the influence of atmospheric conditions. The similarities between the two depths of passive cassette are also observed on the PCA graphs where they are always found in the same group. Also, these results can be explained by fact that coal ash that was filling the passive cassette was somewhat different than the ash currently produced.

Results of five phase sequential extraction indicate possible leaching of As, Ca, Cd, Cu, Zn, and Pb. Among these elements As, Cd, and Pb are toxicologically the most important but they were not present in the first two (easily exchangeable) phases with the exception of As. Arsenic extracted from adsorbed and ion exchange fractions of the active ash cassette indicates a serious environmental problem. Presence of As at least could originate from river water used for transport of ash.

In order to get the basic insight into the data structure and identify similarities and specific grouping patterns, principal component analysis has been carried out on metal content data, for each phase separately. Statistically significant differences were found between metal content of samples from different origins in almost every phase of extraction.

Additional data on the bioavailability of these elements will be obtained by studying their content in plants from these coal ash dumps. These plants should be able to grow under aggravated conditions such as elevated concentration of heavy metals, poor nutrition, and highly alkaline environment. Possibility of their use for phytoremediation will be studied in further research. Various plants will be tested in order to reduce the risk of leaching of toxic contaminants to groundwater, spreading, and erosion.

## Supplementary Material

Table S1. Parameters of descriptive statistics obtained from the mineral content analysis of samples from filter ash.
Table S2. Parameters of descriptive statistics obtained from the mineral content analysis of samples from active cassette.
Table S3. Parameters of descriptive statistics obtained from the mineral content analysis of samples from passive cassette, first depth.
Table S4. Parameters of descriptive statistics obtained from the mineral content analysis of samples from passive cassette, second depth.
Table S5. Results of determination metal concentration in river “Kolubara”.
Table S6. Results of Kruskal-Wallis test.
Table S7. Results of PCA models.
Table S8. Results of determination for each element in reference material ERM CC 141(Loam soil)
Figure S1. The contents of thirteen elements of coal ash by phases.
