# Supplementary material for: Leaching of Major and Minor Elements during the Transport and Storage of Coal Ash Obtained in Power Plant
Source: ScientificWorldJournal. 2014 Jun 29;2014:212506. doi: 10.1155/2014/212506 (PMC4101221; doi:10.1155/2014/212506)
Supplement: Supplementary file 1 — Table S1. Parameters of descriptive statistics obtained from the mineral content analysis of samples from filter ash. Table S2. Parameters of descriptive statistics obtained from the mineral content analysis of samples from active cassette. Table S3. Parameters of descriptive statistics obtained from the mineral content analysis of samples from passive cassette, first depth. Table S4. Parameters of descriptive statistics obtained from the mineral content analysis of samples from passive cassette, second depth. Table S5. Results of determination metal concentration in river “Kolubara”. Table S6. Results of Kruskal-Wallis test. Table S7. Results of PCA models. Table S8. Results of determination for each element in reference material ERM CC 141(Loam soil) Figure S1. The contents of thirteen elements of coal ash by phases. [file 212506.f1.pdf]

Supplementary material

Leaching of major and minor elements during the transport and storage of coal ash obtained in power plant

Authors Rada Krgović[1], Jelena Trifković[2], Dušanka Milojković-Opsenica[2], Dragan Manojlović[2], Jelena Mutić[2]*

[1]*RTB Kolubara, Svetog Save 1, Lazarevac, Serbia*

[2] *Faculty of Chemistry, Studentski trg 12-16, P. O. Box 51, 11158 Belgrade 118, Serbia*

Content

Table S1. Parameters of descriptive statistics obtained from the mineral content analysis of samples from filter ash.

Table S2. Parameters of descriptive statistics obtained from the mineral content analysis of samples from active cassette.

Table S3. Parameters of descriptive statistics obtained from the mineral content analysis of samples from passive cassette, first depth.

Table S4. Parameters of descriptive statistics obtained from the mineral content analysis of samples from passive cassette, second depth.

Table S5. Results of determination metal concentration in river „Kolubara“.

Table S6. Results of Kruskal-Wallis test.

Table S7. Results of PCA models.

Table S8. Results of determination for each element in reference material ERM CC 141(Loam soil)

Figure S1. The contents of thirteen elements of coal ash by phases.

Table S1. Parameters of descriptive statistics obtained from the mineral content analysis of samples from filter ash.

Parameter		I phase	II phase	III phase	IV phase	V phase
As (mg/kg)	Mean	1.20	8.03	187.76	0.99	0.54
	SD	1.26	0.41	4.53	0.98	0.19
	Median	0.65	8.14	188.23	0.85	0.58
	Maximum	4.37	8.68	193.00	3.59	0.79
	Minimum	0.40	7.23	179.65	0.08	0.06
Al (mg/kg)	Mean	34.63	4.63	3336.10	592.75	1386.42
	SD	27.84	1.65	300.42	291.01	122.88
	Median	24.27	4.27	3263.85	503.15	1405.48
	Maximum	93.40	8.38	3906.37	1098.83	1526.13
	Minimum	6.80	2.85	2936.32	235.76	1139.80
Ba (mg/kg)	Mean	34.63	4.25	3291.64	588.86	6.34
	SD	27.84	2.00	279.80	292.18	1.81
	Median	24.27	4.14	3235.72	483.70	6.70
	Maximum	93.40	8.38	3906.37	1098.83	7.96
	Minimum	6.80	1.62	2936.32	235.76	1.73
Ca (mg/kg)	Mean	nd	nd	3.00	nd	1038.61
	SD	nd	nd	3.54	nd	54.55
	Median	nd	nd	1.96	nd	1048.28
	Maximum	nd	nd	9.88	nd	1115.15
	Minimum	nd	nd	nd	nd	960.94
Cd (mg/kg)	Mean	0.02	0.13	0.29	0.05	nd
	SD	0.02	0.01	0.14	0.05	nd
	Median	0.01	0.13	0.24	0.05	nd
	Maximum	0.06	0.15	0.67	0.14	nd
	Minimum	nd	0.12	0.20	nd	nd
Co (mg/kg)	Mean	nd	0.68	5.67	1.80	0.34
	SD	nd	0.03	0.61	0.39	0.05
	Median	nd	0.67	5.73	1.71	0.33
	Maximum	nd	0.73	6.56	2.60	0.42
	Minimum	nd	0.65	4.66	1.38	0.27
Cr (mg/kg)	Mean	2.06	0.77	26.34	15.56	2.28
	SD	0.07	0.04	4.03	2.90	0.17
	Median	2.06	0.77	26.74	15.34	2.28
	Maximum	2.19	0.82	33.31	20.87	2.56
	Minimum	1.95	0.72	19.34	11.11	2.07
Cu (mg/kg)	Mean	0.19	3.79	25.73	8.14	0.08
	SD	0.21	5.54	2.25	1.11	0.03
	Median	0.11	1.20	25.85	8.07	0.08
	Maximum	0.53	17.15	28.79	10.51	0.16
	Minimum	nd	1.11	22.04	6.55	0.04
Fe	Mean	0.07	0.91	6726.34	2257.76	815.48

(mg/kg)	SD	nd	0.41	862.07	458.98	60.49
	Median	0.07	1.04	6675.35	2100.90	807.08
	Maximum	0.07	1.49	7834.17	3237.52	892.72
	Minimum	0.07	0.25	5264.77	1775.12	709.70
Mg (mg/kg)	Mean	52.42	0.04	0.04	0.04	152.50
	SD	40.48	0.00	0.00	0.00	10.66
	Median	43.18	0.04	0.04	0.04	151.65
	Maximum	110.41	0.04	0.04	0.04	165.78
	Minimum	6.93	0.04	0.04	0.04	129.10
Ni (mg/kg)	Mean	nd	nd	nd	nd	1.52
	SD	nd	nd	nd	nd	0.32
	Median	nd	nd	nd	nd	1.61
	Maximum	nd	nd	nd	nd	1.81
	Minimum	nd	nd	nd	nd	0.66
Pb (mg/kg)	Mean	nd	0.41	3.83	0.44	2.20
	SD	nd	0.41	1.76	1.37	0.57
	Median	nd	0.23	3.69	0.00	2.40
	Maximum	nd	1.29	7.61	4.36	2.60
	Minimum	nd	0.06	1.65	0.00	0.62
Zn (mg/kg)	Mean	1.06	5.02	21.68	6.27	nd
	SD	1.27	4.36	1.89	1.82	nd
	Median	0.81	3.04	22.28	5.80	nd
	Maximum	4.10	15.21	24.48	10.32	nd
	Minimum	0.00	2.74	18.76	4.47	nd

nd (not detected, below detection limit of 0.05 μg/g)

Table S2. Parameters of descriptive statistics obtained from the mineral content analysis of samples from active cassette.

Parameter		I phase	II phase	III phase	IV phase	V phase
As (mg/kg)	Mean	18.51	58.94	267.77	0.05	0.71
	SD	5.88	86.14	168.48	0.05	0.48
	Median	17.34	22.92	175.49	0.04	0.59
	Maximum	29.43	287.55	550.62	0.13	1.56
	Minimum	10.35	6.02	110.96	0.00	0.12
Al (mg/kg)	Mean	18.12	5.48	3371.55	626.82	1148.91
	SD	7.41	2.81	371.62	333.12	143.75
	Median	16.30	5.43	3483.18	509.78	1118.85
	Maximum	27.45	11.51	3752.77	1107.42	1386.22
	Minimum	7.03	2.29	2611.33	285.64	967.24
Ba (mg/kg)	Mean	2.33	71.64	90.45	57.41	5.44
	SD	1.13	24.71	42.40	16.19	2.67
	Median	2.39	57.95	84.27	53.34	5.04
	Maximum	3.84	99.95	155.36	84.02	9.54
	Minimum	0.82	45.14	43.07	29.58	1.41
Ca (mg/kg)	Mean	nd	nd	4.53	nd	1022.69
	SD	nd	nd	0.62	nd	452.24
	Median	nd	nd	4.33	nd	783.30
	Maximum	nd	nd	6.19	nd	1778.42
	Minimum	nd	nd	4.05	nd	697.50
Cd (mg/kg)	Mean	0.08	0.35	0.73	0.05	nd
	SD	0.03	0.34	0.68	0.05	nd
	Median	0.08	0.19	0.67	0.04	nd
	Maximum	0.12	1.26	1.78	0.13	nd
	Minimum	0.05	0.13	0.01	0.00	nd
Co (mg/kg)	Mean	0.07	0.35	0.64	0.05	0.21
	SD	0.03	0.34	0.71	0.05	0.09
	Median	0.08	0.19	0.26	0.04	0.17
	Maximum	0.12	1.26	1.78	0.13	0.35
	Minimum	0.01	0.13	0.01	0.00	0.11
Cr (mg/kg)	Mean	0.04	0.17	23.77	14.80	1.70
	SD	0.03	0.06	6.00	3.42	0.48
	Median	0.03	0.16	22.21	14.60	1.59
	Maximum	0.09	0.26	35.29	19.94	2.31
	Minimum	0.01	0.10	16.17	10.49	0.89
Cu (mg/kg)	Mean	2.85	0.84	25.35	9.85	0.09
	SD	1.60	0.73	9.91	2.31	0.13
	Median	2.61	0.70	23.96	9.07	0.00
	Maximum	6.05	2.84	38.38	15.11	0.29

	Minimum	0.33	0.33	13.66	7.48	0.00
Fe (mg/kg)	Mean	10.12	0.96	7487.29	5433.25	663.09
	SD	2.67	0.92	1115.21	9958.56	129.09
	Median	10.04	0.70	7502.97	2326.78	628.66
	Maximum	15.12	3.18	9169.36	33746.90	885.54
	Minimum	4.38	0.16	5694.91	1691.32	519.51
Mg (mg/kg)	Mean	193.52	0.04	0.04	0.04	122.28
	SD	61.36	0.00	0.00	0.00	26.66
	Median	193.64	0.04	0.04	0.04	111.26
	Maximum	279.50	0.04	0.04	0.04	168.03
	Minimum	115.72	0.04	0.04	0.04	100.12
Ni (mg/kg)	Mean	112.80	0.40	nd	nd	1.09
	SD	8.91	0.54	nd	nd	0.30
	Median	113.75	0.00	nd	nd	0.98
	Maximum	122.90	1.31	nd	nd	1.52
	Minimum	96.46	0.00	nd	nd	0.76
Pb (mg/kg)	Mean	1.07	0.13	4.41	0.25	1.81
	SD	0.90	0.10	2.08	0.17	0.62
	Median	0.93	0.10	4.54	0.27	1.76
	Maximum	3.10	0.30	8.36	0.49	2.62
	Minimum	0.15	0.02	1.75	0.02	0.66
Zn (mg/kg)	Mean	0.30	2.29	23.86	8.43	0.00
	SD	0.33	1.08	7.25	1.87	0.01
	Median	0.20	2.19	20.96	9.34	0.00
	Maximum	1.17	4.70	34.16	10.18	0.02
	Minimum	0.02	1.14	16.01	4.28	0.00

nd (not detected, below detection limit of 0.05 µg/g)

Table S3. Parameters of descriptive statistics obtained from the mineral content analysis of samples from passive cassette, first depth.

Parameter		I phase	II phase	III phase	IV phase	V phase
As (mg/kg)	Mean	3.09	10.62	20.14	6.76	nd
	SD	0.37	4.74	4.60	1.57	nd
	Median	3.18	10.43	20.03	7.25	nd
	Maximum	3.46	18.22	27.02	8.46	nd
	Minimum	2.31	1.01	13.41	3.45	nd
Al (mg/kg)	Mean	17.56	793.75	3172.83	648.43	468.00
	SD	10.24	296.58	435.69	69.41	98.00
	Median	14.20	857.78	3282.52	643.53	487.65
	Maximum	38.95	1141.99	3707.98	772.64	571.54
	Minimum	5.71	0.80	2549.25	547.68	235.99
Ba (mg/kg)	Mean	2.33	74.82	90.96	31.08	1.61
	SD	1.12	24.99	42.71	4.38	0.69
	Median	2.35	75.32	82.25	31.51	1.74
	Maximum	3.92	103.53	158.32	36.96	2.73
	Minimum	0.85	43.07	40.67	24.21	0.39
Ca (mg/kg)	Mean	nd	nd	4.56	nd	507.62
	SD	nd	nd	0.60	nd	115.56
	Median	nd	nd	4.41	nd	496.91
	Maximum	nd	nd	6.06	nd	769.44
	Minimum	nd	nd	3.98	nd	348.15
Cd (mg/kg)	Mean	0.03	0.20	nd	0.06	0.01
	SD	0.02	0.06	nd	0.03	0.00
	Median	0.02	0.22	nd	0.07	0.01
	Maximum	0.09	0.27	nd	0.10	0.02
	Minimum	0.02	0.03	nd	0.00	0.01
Co (mg/kg)	Mean	nd	1.97	2.95	2.14	0.10
	SD	nd	0.26	0.81	0.26	0.07
	Median	nd	1.95	2.82	2.18	0.12
	Maximum	nd	2.42	4.42	2.65	0.20
	Minimum	nd	1.54	1.85	1.72	0.00
Cr (mg/kg)	Mean	0.06	8.61	13.67	20.91	0.11
	SD	0.03	1.43	2.93	3.01	0.04
	Median	0.05	8.56	12.96	21.84	0.13
	Maximum	0.14	11.17	19.67	23.78	0.15
	Minimum	0.04	6.60	9.82	14.68	0.04
Cu (mg/kg)	Mean	0.12	5.79	15.90	8.93	nd
	SD	0.06	2.21	3.79	2.45	nd
	Median	0.13	5.99	15.46	8.12	nd
	Maximum	0.23	8.94	25.32	14.58	nd

	Minimum	0.03	0.71	10.90	6.40	nd
Fe (mg/kg)	Mean	6.18	1863.38	3460.59	2953.95	395.71
	SD	8.70	701.04	815.05	332.95	114.99
	Median	1.06	2108.69	3332.82	3036.54	441.46
	Maximum	24.63	2397.93	5410.24	3399.96	511.29
	Minimum	0.12	0.36	2350.15	2488.86	201.34
Mg (mg/kg)	Mean	109.74	0.04	0.04	0.04	67.07
	SD	13.56	0.00	0.00	0.00	15.09
	Median	108.76	0.04	0.04	0.04	73.38
	Maximum	132.92	0.04	0.04	0.04	79.05
	Minimum	93.82	0.04	0.04	0.04	31.30
Ni (mg/kg)	Mean	nd	6.82	15.26	12.01	0.98
	SD	nd	2.52	3.81	1.07	0.32
	Median	nd	7.48	15.27	12.33	1.03
	Maximum	nd	9.31	21.89	13.35	1.50
	Minimum	nd	0.28	9.11	9.86	0.48
Pb (mg/kg)	Mean	0.03	2.84	5.70	3.26	1.13
	SD	0.02	1.33	1.02	9.69	0.37
	Median	0.02	2.75	5.55	0.15	1.31
	Maximum	0.07	5.98	7.39	30.84	1.43
	Minimum	0.00	0.80	4.60	0.08	0.19
Zn (mg/kg)	Mean	0.04	8.15	5.89	4.96	nd
	SD	0.07	2.97	1.40	0.61	nd
	Median	0.01	8.48	5.30	4.82	nd
	Maximum	0.21	11.66	9.17	6.32	nd
	Minimum	0.00	0.47	4.26	3.97	nd

nd (not detected, below detection limit of 0.05 µg/g)

Table S4. Parameters of descriptive statistics obtained from the mineral content analysis of samples from passive cassette, second depth.

Parameter		I phase	II phase	III phase	IV phase	V phase
As (mg/kg)	Mean	3.09	8.60	37.19	5.55	0.02
	SD	0.31	6.02	30.35	2.44	0.04
	Median	3.05	11.31	26.89	5.82	0.01
	Maximum	3.81	15.48	120.40	10.56	0.13
	Minimum	2.76	1.15	19.14	1.90	0.00
Al (mg/kg)	Mean	44.89	322.52	3163.95	736.49	559.42
	SD	41.02	267.51	567.59	178.40	70.01
	Median	21.02	522.40	3175.00	651.81	572.80
	Maximum	123.56	541.89	3921.80	1057.87	646.43
	Minimum	14.33	7.28	2527.21	553.30	419.93
Ba (mg/kg)	Mean	0.73	81.81	28.48	28.37	2.32
	SD	0.20	32.07	6.84	4.56	0.54
	Median	0.70	102.33	27.43	27.89	2.11
	Maximum	1.04	111.16	43.96	35.47	3.33
	Minimum	0.44	40.60	20.01	22.33	1.67
Ca (mg/kg)	Mean	0.01	0.01	9.35	0.01	622.60
	SD	0.00	0.00	6.10	0.00	192.88
	Median	0.01	0.01	7.20	0.01	528.00
	Maximum	0.01	0.01	24.51	0.01	900.59
	Minimum	0.01	0.01	4.66	0.01	403.34
Cd (mg/kg)	Mean	0.03	0.17	nd	0.09	0.01
	SD	0.02	0.07	nd	0.05	0.00
	Median	0.02	0.21	nd	0.08	0.01
	Maximum	0.05	0.23	nd	0.19	0.01
	Minimum	0.01	0.06	nd	0.03	0.00
Co (mg/kg)	Mean	0.01	1.30	2.93	5.99	0.14
	SD	0.01	1.05	0.47	0.83	0.04
	Median	0.01	2.06	2.77	5.74	0.13
	Maximum	0.04	2.16	3.57	7.93	0.20
	Minimum	0.00	0.07	2.20	5.17	0.09
Cr (mg/kg)	Mean	0.14	5.38	13.09	17.57	1.20
	SD	0.15	4.62	2.27	2.41	0.17
	Median	0.04	7.90	12.98	16.81	1.16
	Maximum	0.39	9.78	18.91	21.11	1.54
	Minimum	0.03	0.05	10.67	14.43	0.98
Cu (mg/kg)	Mean	0.22	3.65	14.98	7.88	nd
	SD	0.16	2.85	3.17	0.79	nd
	Median	0.14	5.40	13.90	7.92	nd
	Maximum	0.51	6.69	22.02	8.74	nd

	Minimum	0.09	0.12	11.01	6.07	nd
Fe (mg/kg)	Mean	19.12	1178.42	4037.72	2627.41	466.42
	SD	24.77	1022.92	664.04	294.59	59.08
	Median	0.89	1731.39	3813.01	2636.26	461.59
	Maximum	59.66	2247.15	5171.57	3025.83	557.83
	Minimum	0.19	1.68	3334.01	2104.00	379.97
Mg (mg/kg)	Mean	81.21	0.04	0.04	0.04	73.10
	SD	10.28	0.00	0.00	0.00	9.29
	Median	84.17	0.04	0.04	0.04	74.85
	Maximum	92.96	0.04	0.04	0.04	86.52
	Minimum	61.28	0.04	0.04	0.04	58.57
Ni (mg/kg)	Mean	nd	4.91	17.58	12.38	1.07
	SD	nd	3.74	3.48	1.81	0.14
	Median	nd	7.54	16.03	12.04	1.00
	Maximum	nd	8.28	22.74	15.35	1.29
	Minimum	nd	0.48	14.25	10.10	0.92
Pb (mg/kg)	Mean	0.07	1.52	4.52	0.20	1.32
	SD	0.04	1.27	1.28	0.13	0.10
	Median	0.05	2.28	4.92	0.20	1.29
	Maximum	0.14	2.91	6.00	0.41	1.48
	Minimum	0.04	0.01	2.34	0.00	1.20
Zn (mg/kg)	Mean	0.27	4.53	7.70	12.91	nd
	SD	0.53	2.54	2.39	23.69	nd
	Median	0.08	5.92	6.50	5.26	nd
	Maximum	1.74	7.19	11.50	80.19	nd
	Minimum	0.00	0.78	5.07	4.28	nd

nd (not detected, below detection limit of 0.05 µg/g)

Table S5. Results of determination metal concentration in river „Kolubara“

Parameter	I quartal	II quartal	III quartal	IV quartal
Mg, mg/L	11.8	12.3	12.4	11.4
Ca, mg/L	62.7	66.9	65.6	54.3
Fe, mg/L	0.4	0.3	0.2	0.2
Al, mg/L	1.30	0.4	0.33	0.39
Cu, mg/L	0.1	0.1	0.1	0.1
Ni, µg/L	n.d.	10	10	10
As, µg/L	n.d.	**6.87**	n.d.	n.d.
Cd, µg/L	3	1.1	n.d.	n.d.
Cr, µg/L	30	7.5	6.0	5.58
Co, µg/L	18	n.d.	n.d.	n.d.

nd (not detected, below detection limit of 1 µg/L)

Table S6. Results of Kruskal-Wallis test.

**Parameter**		χ^{2a}	**P**	**KW Multiple comparison Z-Value Test**[b]
Al	I phase	6.64	0.0843	Pa (Pb)
	II phase	24.06	<0.0001	A (Pa, Pb)<br>EF (Pa, Pb)<br>Pa (A, EF)<br>Pb (A, EF)
	III phase	1.53	0.6755	-
	IV phase	3.24	0.3561	-
	V phase	24.02	<0.0001	A (Pa)<br>EF (Pa, Pb)<br>Pa (A, EF)<br>Pb (EF)
As	I phase	29.03	<0.0001	A (EF, Pa, Pb)<br>EF (A, Pa, Pb)<br>Pa (A, EF)<br>Pb (A, EF)
	II phase	13.91	0.0030	A (EF, Pa, Pb)<br>EF (A)<br>Pa (A)<br>Pb (A)
	III phase	30.25	<0.0001	A (Pa, Pb)<br>EF (Pa, Pb)<br>Pa (A, EF)<br>Pb (A, EF)
	IV phase	32.61	<0.0001	A (Pa, Pb)<br>EF (Pa, Pb)<br>Pa (A, EF)<br>Pb (A, EF)
	V phase	20.53	0.0001	A (Pa, Pb)<br>EF (Pa, Pb)<br>Pa (A, EF)<br>Pb (A, EF)
Ba	I phase	31.44	<0.0001	A (EF, Pb)<br>EF (A, Pa, Pb)<br>Pa (EF, Pb)<br>Pb (A, EF, Pa)
	II phase	21.26	0.0001	A (EF)<br>EF (A, Pa, Pb)<br>Pa (EF)<br>Pb (EF)
	III phase	32.49	<0.0001	A (EF, Pb)<br>EF (A, Pa, Pb)<br>Pa (EF, Pb)<br>Pb (A, EF, Pa)
	IV phase	31.49	<0.0001	A (EF, Pa, Pb)<br>EF (A, Pa, Pb)<br>Pa (A, EF)<br>Pb (A, EF)
	V phase	17.16	0.0007	A (Pa)<br>EA (Pa, Pb)<br>Pa (A, EF)<br>Pb (EF)
Ca	III phase	16.08	0.0011	A (Pb)<br>EF (Pb)

	V phase	18.31	0.0004	Pa (Pb)<br>Pb (A, EF, Pa)<br>A (Pa, Pb)<br>EF (Pa, Pb)<br>Pa (A, EF)<br>Pb (A, EF)
Cd	I phase	22.19	<0.0001	A (EF, Pa, Pb)<br>EF (A, Pa)<br>Pa (A, EF)<br>Pb (A)
	II phase	12.19	0.0068	A (EF)<br>EF (A, Pa)<br>Pa (EF)
	III phase	29.39	<0.0001	A (Pa, Pb)<br>EF (Pa, Pb)<br>Pa (A, EF)<br>Pb (A, EF)
	IV phase	4.88	0.1805	EA (Pb)<br>Pb (EA)
	V phase	8.48	0.03706	A (EF)<br>EF (A, Pa, Pb)<br>Pa (EF)<br>Pb (EF)
Co	I phase	30.46	<0.0001	A (EF, Pa, Pb)<br>EF (A, Pa, Pb)<br>Pa (A, EF)<br>Pb (A, EF)
	II phase	15.97	0.0011	A (Pa, Pb)<br>EF (Pa)<br>Pa (A, EF)<br>Pb (A)
	III phase	32.93	<0.0001	A (EF, Pa, Pb)<br>EF (A, Pa, Pb)<br>Pa (A, EF)<br>Pb (A, EF)
	IV phase	34.16	<0.0001	A (EF, Pa, Pb)<br>EF (A, Pb)<br>Pa (A, Pb)<br>Pb (A, Pa)
	V phase	16.00	0.0011	A (EF)<br>EF (A, Pa, Pb)<br>Pa (EF)<br>Pb (EF)
Cr	I phase	24.79	<0.0001	A (EF)
	II phase	18.46	0.0003	A (Pa, Pb)<br>EF (Pa)<br>Pa (A, EF, Pb)<br>Pb (A, Pa)
	III phase	27.87	<0.0001	A (Pa, Pb)<br>EF (Pa, Pb)<br>Pa (A, EF)<br>Pb (A, EF)
	IV phase	16.07	0.0011	A (Pa)<br>EF (Pa)<br>Pa (A, EF)
	V phase	23.43	<0.0001	A (Pa)<br>EF (Pa, Pb)<br>Pa (A, EF)<br>Pb (EF)
Cu	I phase	21.47	<0.0001	A (EF, Pa, Pb)

				EF (A)<br>Pa (A)<br>Pb (A)
	II phase	14.40	0.0024	A (EF, Pa, Pb)<br>EF (A)<br>Pa (A)<br>Pb (A)
	III phase	20.99	0.0001	A (Pa, Pb)<br>EF (Pa, Pb)<br>Pa (A, EF)<br>Pb (A, EF)
	IV phase	5.90	0.1163	A (Pb)<br>Pb (A)
	V phase	12.12	0.0070	EF (Pa, Pb)<br>Pa (EF)<br>Pb (EF)
Fe	I phase	23.45	<0.0001	A (EF)<br>EF (A, Pa, Pb)<br>Pa (EF)<br>Pb (EF)
	II phase	24.14	<0.0001	A (Pa, Pb)<br>EF (Pa, Pb)<br>Pa (A, EF)<br>Pb (A, EF)
	III phase	30.83	<0.0001	A (Pa, Pb)<br>EF (Pa, Pb)<br>Pa (A, EF)<br>Pb (A, EF)
	IV phase	11.47	0.0094	A (Pa)<br>EF (Pa)<br>Pa (A, EF)
	V phase	21.79	<0.0001	A (Pa, Pb)<br>EF (Pa, Pb)<br>Pa (A, EF)<br>Pb (A, EF)
Mg	I phase	29.93	<0.0001	A (EF, Pb)<br>EF (A, Pa)<br>Pa (EF, Pb)<br>Pb (A, Pa)
	V phase	21.67	<0.0001	A (Pa, Pb)<br>EF (Pa, Pb)<br>Pa (A, EF)<br>Pb (A, EF)
Ni	I phase	21.95	<0.0001	A (EF, Pa, Pb)<br>EF (A)<br>Pa (A)<br>Pb (A)
	II phase	25.27	<0.0001	A (Pa, Pb)<br>EF (Pa, Pb)<br>Pa (A, EF)<br>Pb (A, EF)
	III phase	29.69	<0.0001	A (Pa, Pb)<br>EF (Pa, Pb)<br>Pa (A, EF)<br>Pb (A, EF)
	IV phase	29.30	<0.0001	A (Pa, Pb)<br>EF (Pa, Pb)<br>Pa (A, EF)<br>Pb (A, EF)
	V phase	14.07	0.0028	A (EF)

				EF (A, Pa, Pb)<br>Pa (EF)<br>Pb (EF)
Pb	I phase	34.82	<0.0001	A (EF, Pa, Pb)<br>EF (A, Pa, Pb)<br>Pa (A, EF)<br>Pb (A, EF)
	II phase	18.88	0.0003	A (Pa, Pb)<br>EF (Pa)<br>Pa (A, EF)<br>Pb (A, Pa)
	III phase	7.11	0.0683	EF (Pa)<br>Pa (EF)
	IV phase	12.08	0.0071	A (EF)<br>EF (A, Pa, Pb)<br>Pa (EF)<br>Pb (EF)
	V phase	17.50	0.0006	A (Pa)<br>EF (Pa, Pb)<br>Pa (A, EF)<br>Pb (EF)
Zn	I phase	10.16	0.0173	A (Pa)<br>EF (Pa)<br>Pa (A, EF)
	II phase	15.13	0.0017	A (EF, Pa)<br>EF (A)<br>Pa (A, Pb)<br>Pb (Pa)
	III phase	30.19	<0.0001	A (Pa, Pb)<br>EF (Pa, Pb)<br>Pa (A, EF)<br>Pb (A, EF)
	IV phase	11.04	0.0115	A (Pa, Pb)<br>Pa (A)<br>Pb (A)
	V phase	0.31	0.9581	-

[a] $\chi^2_{cr(df(3);\alpha=0.05)} = 7.81$

[b]Regular test: Medians significantly different if z-value > 1.9600

Table S7. Results of PCA models.

PCA model	No of PCs	% Variance captured by PC1	% Variance captured by PC2	% Variance captured by PC3
I phase	3	47.84	15.51	14.07
II phase	3	62.79	19.49	10.06
III phase	6	49.83	15.74	10.36
IV phase	3	31.17	16.16	13.15
V phase	4	73.33	12.07	5.10

Table S8. Results of determination for each element in reference material ERM CC 141(Loam soil)

Aqua regia extractable content	ERM CC 141 Loam soil	
	Certified value ± uncertainty* (mg/kg)	Found value ± uncertainty (mg/kg)
As	7.5 ± 1.4	7.9 ± 0.6
Cd	0.25 ± 0.04	0.30 ± 0.04
Co	7.9 ± 0.9	8.0 ± 0.2
Cr	31 ± 4	33.2 ± 0.2
Cu	12.4 ± 0.9	12.9 ± 0.3
Zn	50 ± 4	50.3 ± 0.7
Ni	21.9 ± 1.6	23.0 ± 0.2
Pb	32.2 ± 1.4	31.5 ± 0.5
Major components	Additional material information (g/kg)	Found value (g/kg)
Al_2O_3**	167.0	170.8
Fe_2O_3 **	65.8	66.0
CaO**	6.2	5.8
Mg**	6.8	5.9

*uncertainty for 95 % confidence level (coverage factor k=2)

**Obtained by k_o-neuton activation analysis

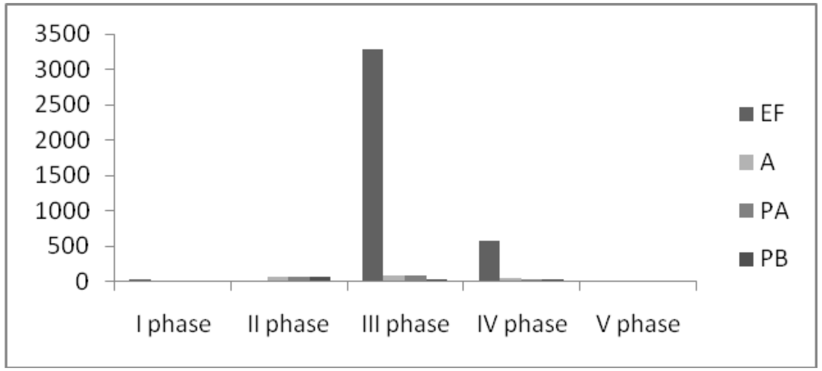

Ba

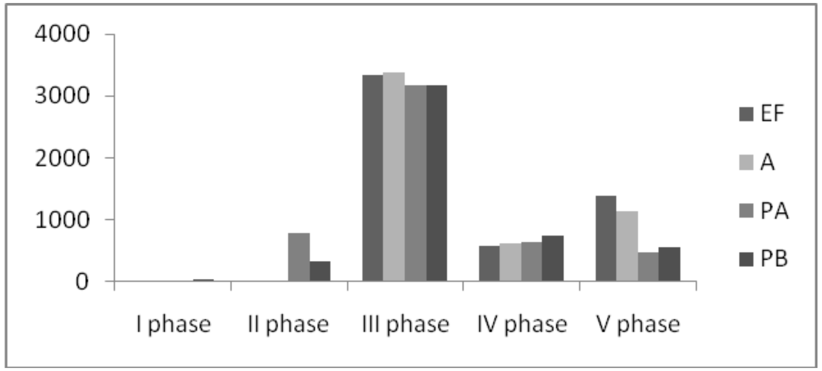

Al

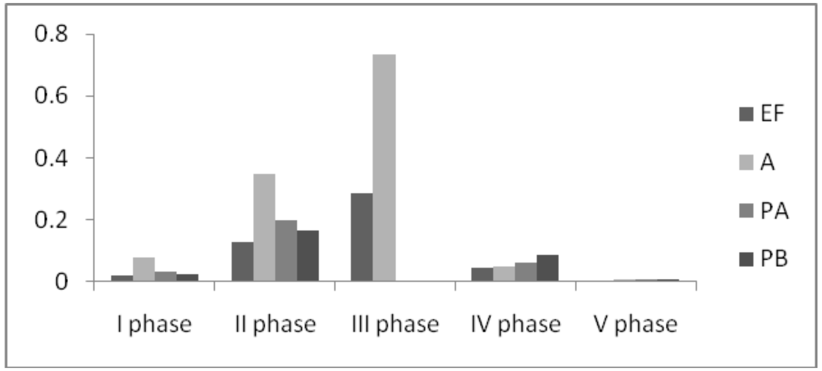

Cd

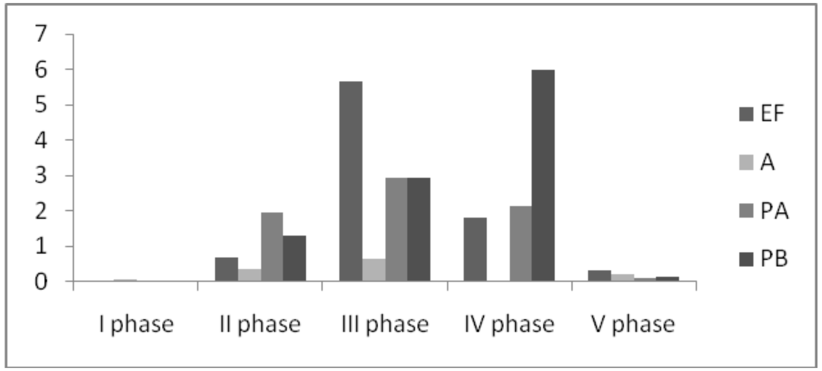

Co

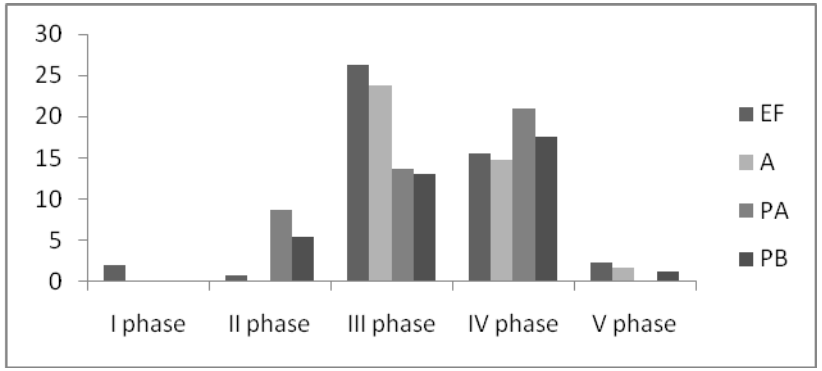

Cr

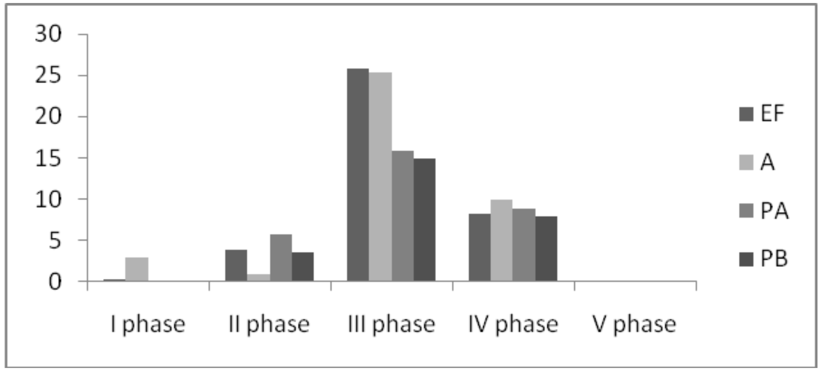

Cu

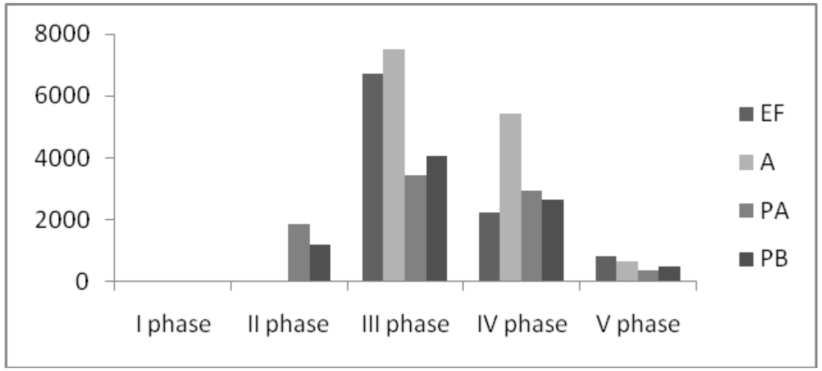

Fe

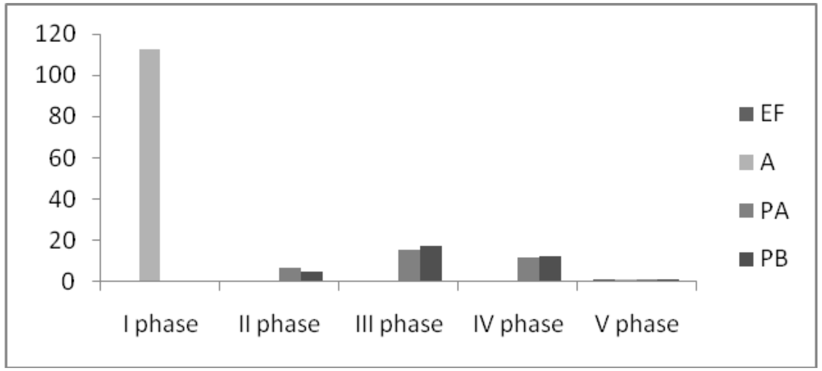

Ni

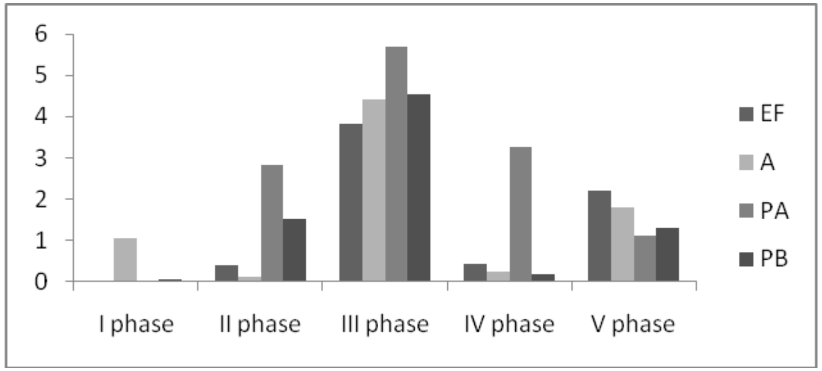

Pb

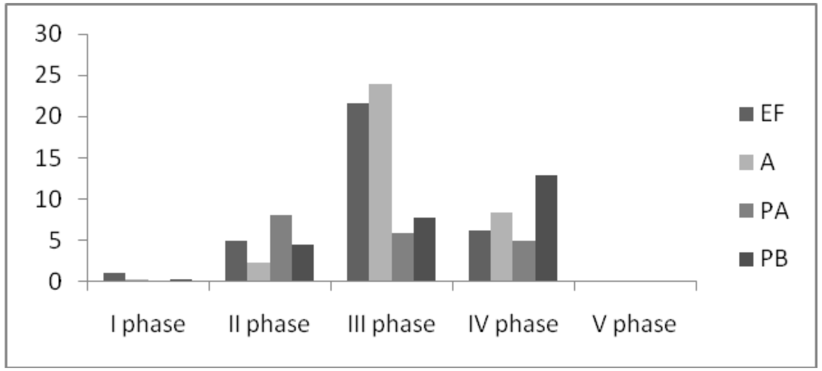

Zn

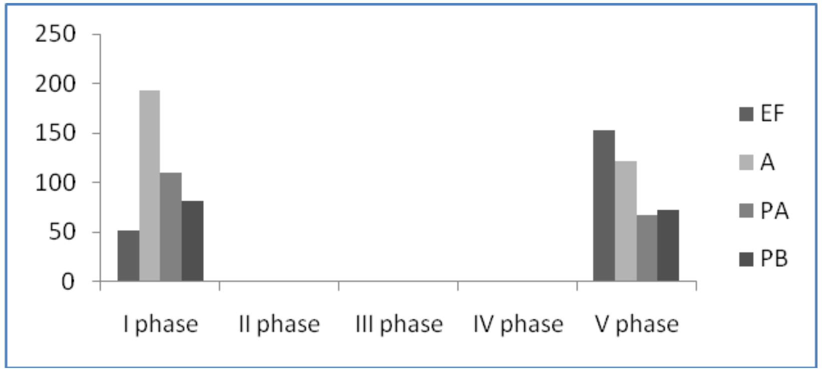

Mg

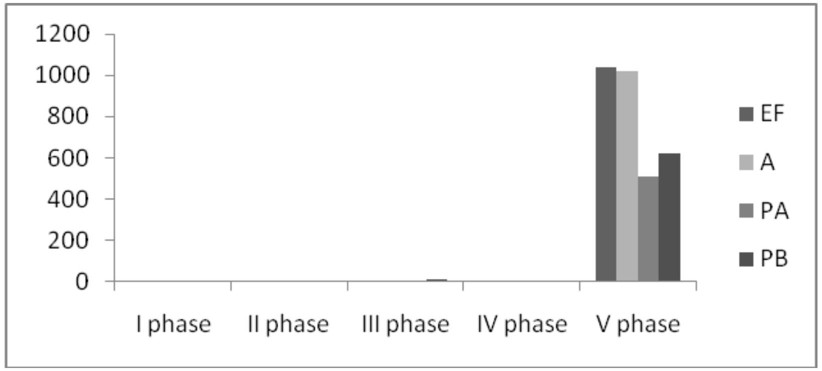

Ca

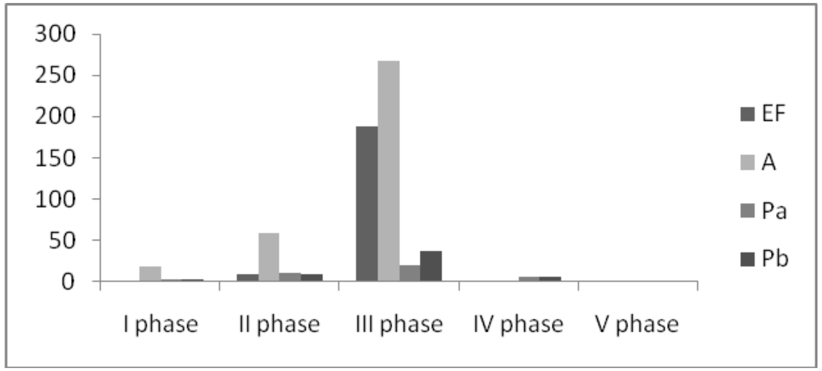

As

Figure S1. The contents of thirteen elements of coal ash by phases.
